# Flexible hydrogen-bonded organic frameworks (HOFs): opportunities and challenges

**DOI:** 10.1039/d4sc02628d

**Published:** 2024-05-21

**Authors:** Jiantang Li, Banglin Chen

**Affiliations:** a Key Laboratory of the Ministry of Education for Advanced Catalysis Materials, College of Chemistry and Materials Sciences, Zhejiang Normal University Jinhua 321004 P. R. China jiantang.li@zjnu.edu.cn; b Fujian Key Laboratory of Polymer Materials, College of Chemistry and Materials Sciences, Fujian Normal University Fujian 350007 P. R. China banglin.chen@fjnu.edu.cn

## Abstract

Flexible behavior is one of the most fascinating features of hydrogen-bonded organic frameworks (HOFs), which represent an emerging class of porous materials that are self-assembled *via* H-bonding between organic building units. Due to their unique flexibility, HOFs can undergo structural changes or transformations in response to various stimuli (physical or chemical). Taking advantage of this unique structural feature, flexible HOFs show potential in multifunctional applications such as gas storage/separation, molecular recognition, sensing, proton conductivity, biomedicine, *etc.* While some other flexible porous materials have been extensively studied, the dynamic behavior of HOFs remains relatively less explored. This perspective highlights the inherent flexible properties of HOFs, discusses their different flexible behaviors, including pore size/shape changes, interpenetration/stacking manner, H-bond breaking/reconstruction, and local dynamic behavior, and highlights their potential applications. We believe that this perspective will not only contribute to HOF chemistry and materials science, but will also facilitate the ongoing extensive research on dynamic porous materials.

## Abstract 2

In this perspective, we have discussed the recent progress of flexible HOFs, with particular focus on various dynamic behaviors and their applications in different fields. Finally, the current challenges and opportunities in this field are discussed.

## Graphical abstract

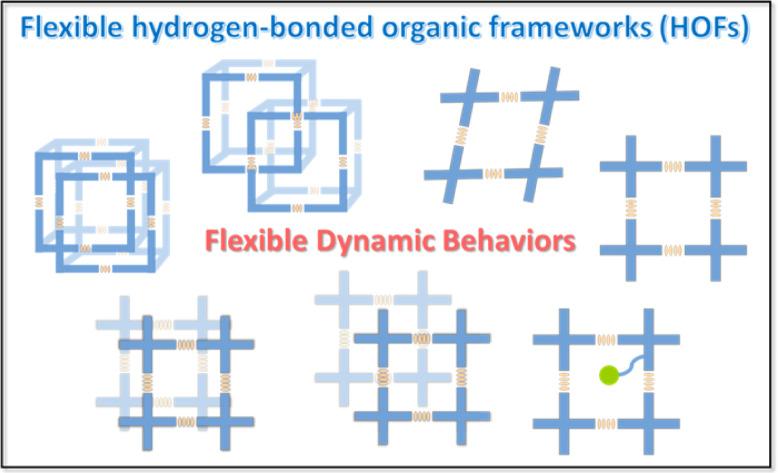

## 1. Introduction

Porous solids, which include zeolites, metal–organic frameworks (MOFs), covalent-organic frameworks (COFs), *etc.*, have entered a whole new era along with the rapid development of reticular chemistry. The research of porous materials constructed *via* strong bonds (coordination bonds and covalent bonds) has advanced our understanding of functional-directed design, and the development of various synthetic tools has enriched the library of reticular chemistry. These materials possess tunable porosity and adjustable pore structures, enabling high customization and functionality, thus emerging as one of the most rapidly growing fields in chemistry and materials science.^1–9^

Hydrogen-bonded organic frameworks (HOFs), a novel class of porous materials, are formed by the self-assembly of organic building units *via* intermolecular H-bonding interactions. Unlike MOFs and COFs, which are constructed through strong coordination or covalent bonds, the weaker bond strength and flexible feature of H-bonds lead to challenges in terms of the stability and structural design of HOFs, but at the same time, bring about new opportunities for structural diversity and flexibility.^10–22^ In recent years, HOFs have shown great potential in gas storage/separation, sensing, proton conduction, *etc.*

**Fig. 1:**
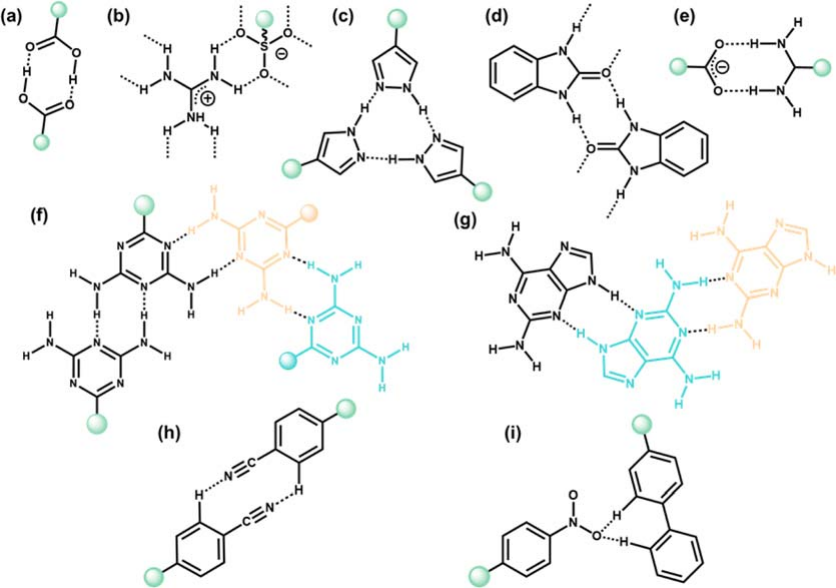
Representative H-bonding motifs for the design of HOFs: (a) carboxyl dimer; (b) guanidinium-sulfonate; (c) pyrazole; (d) benzimidazolone; (e) amidinium-carboxylate; (f) 2,4-diaminotriazine; (g) 2,6-diaminopurine; (h) cyano group; (i) nitro group.

H-bonds are a type of weak supramolecular interaction characterized by an attractive force between permanent dipoles, occurring between a hydrogen atom bonded to another atom *via* a covalent bond and another electronegative atom (X–H⋯Y). Typically, the atoms (X and Y) on both sides of the hydrogen atom involved in H-bonding are highly electronegative (*e.g.*, N and O). It's worth noting that H-bonds can form both intermolecularly and intramolecularly. Although many arguments exist about H-bonds, their widespread presence and significance in chemistry, materials science, and biology have been recognized. As shown in Fig. 1, some common H-bond motifs used for constructing HOFs include carboxyl dimer, guanidinium-sulfonate, pyrazole, benzimidazolone, amidinium-carboxylate, 2,4-diaminotriazine, 2,6-diaminopurine, *etc.* For these typical H-bonds, the bond lengths (X–H⋯Y, distance between X and Y) usually range from 2.5 to 3.2 Å, much longer than the 1.2–1.5 Å typically found in covalent bonds. Therefore, the flexibility of H-bonding is one of its essential features in terms of strength and angle.

Open frameworks based on H-bonds have been reported as early as 1914,^23^ and along with the development of single-crystal diffraction techniques, many related structures have been published since then. However, most of them have remained at the level of structural characterization; this may be due to the lack of in-depth understanding and effective activation methods in the early days, similar to the challenges faced in the early development of MOFs.^24^ In 2011, Chen *et al.* successfully achieved the activation of HOF-1, a compound that was reported more than a decade ago *via* a 2,4-diaminotriazine moiety,^25,26^ using a solvent exchange strategy, demonstrating for the first time the permanent porosity and flexibility of this type of material. They also discovered its potential application in gas separation as the first representative of a functional HOF.^27^ In the same period, Schröder *et al.* reported another example of a supramolecular organic framework (SOF-1) with permanent porosity, marking the official beginning of the era of rapid development of HOFs.^28^

**Fig. 2:**
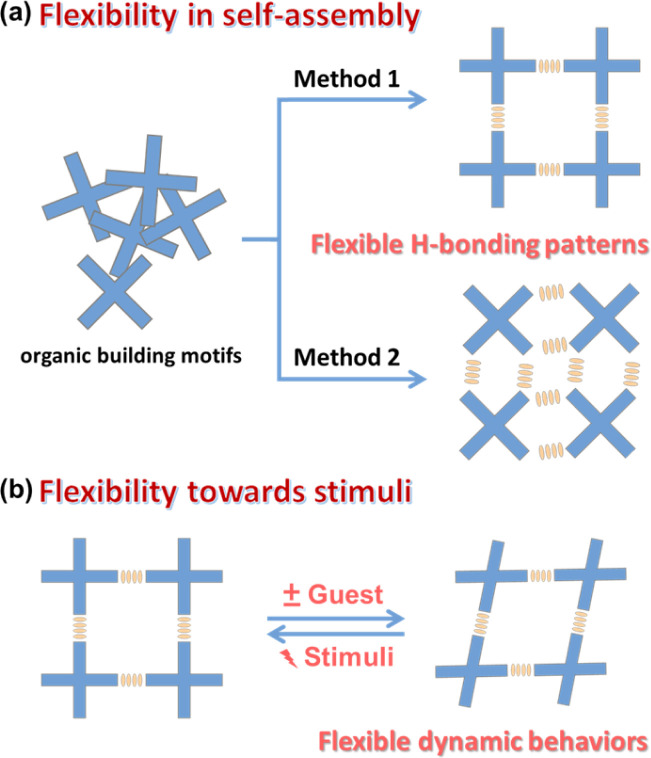
The flexibility of HOFs in (a) the self-assembly process and (b) response to external stimuli.

Flexibility is one of the most fascinating features of porous materials, especially evident in flexible MOFs and COFs.^29–34^ Much of the flexibility of HOFs comes from the weak H-bonding strength and the wide range of H-bond angles (from 130° to 180°). In some cases, this angle even approaches 90°.^11^ Such a flexibility of H-bonding will significantly affect the structural diversity of HOFs. Functional modification of organic motifs is a very effective method to regulate the H-bonding patterns by using steric hindrance or planarity changes. As in the case of HOF-BTB, HOF-22 and PFC-12, the organic units are similar, but the effects of functionalization result in completely different structures.^35–37^ Even for the same organic motifs, changing solvents/conditions could have a considerable impact on H-bonding patterns during crystallization (Fig. 2). For example, the rapid recrystallization of tris(4-carboxyphenyl)amine (TCA) in methanol can result in HOF-16 with free –COOH sites in the channel, whereas it can lead to HOF-11 in THF/hexane with an inert pore surface.^38^ The flexibility in the self-assembly process has greatly increased the diversity of HOFs while posing challenges for targeted synthesis. In 2011, Cooper *et al.* demonstrated that the complicated assembly of porous cages can be precisely predicted by lattice energy calculations, which exhibited the application of “design by computational selection” for porous organic frameworks.^39^ Therefore, scientists have always remained hopeful of controlling the flexible and complex self-assembly behavior and mastering effective strategies for targeting and constructing HOFs. Furthermore, the dynamic behaviors of HOFs in response to external stimuli are also quite attractive. This dynamic behavior can significantly affect the properties of HOFs and even lead to many unpredictable results, which we will discuss in detail in this paper.

Flexible HOFs have some obvious advantages: (1) more accessible flexible behaviors. Generally, the bonding energy of H-bonds is 10–40 kJ mol^−1^, much lower than the coordination bonds in MOFs (90–350 kJ mol^−1^) and the covalent bonds in COFs (300–600 kJ mol^−1^), making it easier for HOFs to exhibit a certain degree of structural flexibility and diversity;^13^ (2) better reproducibility. The reversibility of H-bonds allows us to reconnect broken H-bonding units under certain conditions, enabling the original HOFs to recover and regenerate; (3) compared to MOFs, HOFs lack metal ions (or have a lower proportion of metal ions), resulting in lower density; however they exhibit higher structural diversity than COFs, combining the different advantages of both.

In this perspective, we aim to provide a unique viewpoint on discussing flexible HOFs. Rather than offering a comprehensive overview of all flexible HOFs, we will focus on discussing the flexible behaviors of some representative flexible HOFs (Table 1) and their unique applications. Additionally, we will highlight the differences between flexible HOFs and other porous materials such as MOFs and COFs.

**Table 1:** Selected representative flexible HOFs categorized into different flexible behaviors

Flexible behaviors		HOFs	Functionality	Ref.
Pore size/shape changes	Pore contraction/expansion	SOF-1	C_2_H_2_, CH_4_, CO_2_ storage	28
		HOF-1	C_2_H_2_/C_2_H_4_ separation	27
		HOF-4	C_2_H_4_/C_2_H_6_ separation	40
		ZJU-HOF-1	C_2_H_6_/C_2_H_4_ separation	41
		HOF-40	Xe/Kr separation	42
		HOF-FJU-8	C_2_H_2_/CO_2_ separation	43
		Compound 2	O_2_/Ar/N_2_ separation	44
	Pore deformation	HOF-5	CO_2_/CH_4_ separation	45
			CO_2_/N_2_ separation	
		HOF-7	CO_2_/N_2_ separation	46
		8PN	Molecular recognition	47
		2D-90	Dynamic luminescence behavior	48
Interpenetration/stacking manner	Interpenetrated network	HOF-6	Proton conductivity	49
		PET-HOF-1	—	50
		PET-HOF-2	—	
		HOF-12	CO_2_/CH_4_ separation	51
		HOF-FJU-1	C_2_H_6_/C_2_H_4_ separation	52
			C_3_H_6_/C_3_H_8_ separation	53
			C_2_H_2_/CO_2_ separation	54
		ZJU-HOF-8a	*n*-C_4_H_10_/CH_4_ separation	55
	Layer network sliding	LA-H-HexNet	Hydrocarbon sorption	56
		HOF-29	Molecular recognition	57
		HOF-FJU-88	CO_2_/C_2_H_2_ separation	58
H-bond breaking/reconstruction		[(Bmib)_3_(H_2_O)_12_]_*n*_	—	59
		HOF-30	C_3_H_6_/C_3_H_4_ separation	60
		PFC-71/72/73	Photocatalytic CO_2_ reduction	61
		Tri-PMDI-Br	Bz/Cy separation	62
		MEP-HOF	Detection of nitrobenzene	63
		FDU-HOF-3	Ammonia capture	64
		PFC-77	—	65
		PFC-78	—	
		PFC-79	—	
		HOF-FJU-2	Acetone/methanol separation	66
		HOF-NBDA	C_2_H_6_/C_2_H_4_ separation	67
		BA-N	Detection of acetone	68
		BA-C	—	
		HOF-30	C_3_H_4_/C_3_H_6_ separation	69
		H_c_OF-6	—	70
Local dynamic behavior		ABTPA-1	—	71
		ABTPA-2	—	
		8PZ	Programmable luminescence	72
		Cage-6-COOH	Vapor sorption	73

## 2. Different types of flexible behaviors

**Fig. 3:**
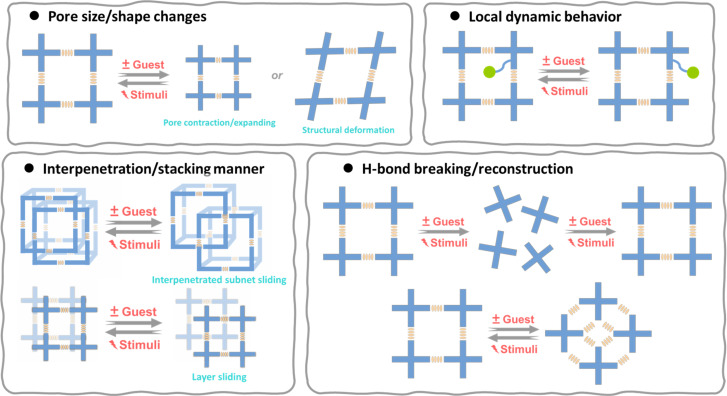
Representative modes of different types of flexible behaviors for flexible HOFs.

The flexibility of HOFs derives from the organic motifs as well as the weak bond strength and reversibility of the H-bond. We have so far obtained a lot of flexible HOFs, although in many cases, the fragility of the crystals makes it challenging to identify the structural changes where the flexible behavior occurs. Generally speaking, during the synthesis or in response to external stimuli, HOFs display four primary flexible features: pore size/shape changes, interpenetration/stacking manner, H-bond breaking/reconstruction, and local dynamic behavior (Fig. 3).

### 2.1 Pore size/shape changes

The removal of guest molecules during the activation of flexible HOFs or under certain external stimuli often causes pore contraction/expansion, deformation of the structure, or both.^27,28,40–48^

#### 2.1.1 Pore contraction/expansion

**Fig. 4:**
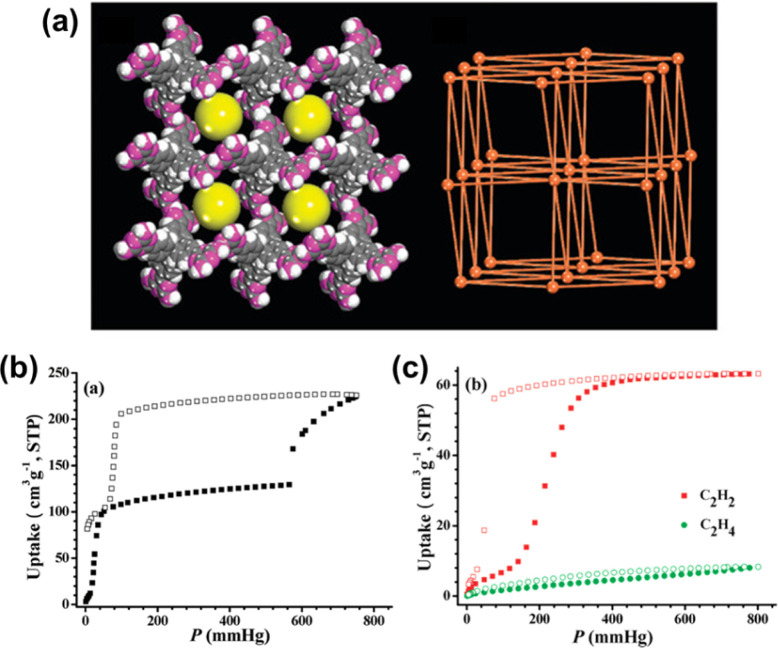
(a) Single-crystal structure and topology of HOF-1; (b) CO_2_ adsorption at 196 K; (c) C_2_H_2_ and C_2_H_4_ adsorption of HOF-1 at 298 K.^27^

HOF-1 is a well-known HOF that first exhibited pore contraction/expansion behavior in gas adsorption.^27^ This HOF is constructed from tetrakis(4-(2,4-diamino-1,3,5-trizenyl)phenyl)methane *via* H-bonds between 2,4-diaminotriazine groups (Fig. 4a). The CO_2_ adsorption at 196 K shows an obvious gate-opening effect at *P*/*P*_0_ = 0.78 as well as a large hysteretic desorption behavior, indicating that the pore structure of activated HOF-1a expanded with the increase of pressure to adsorb more guest molecules (Fig. 4b). Similar dynamic behavior was also observed for C_2_H_2_ (Fig. 4c). PXRD confirms the reversible transformation between the guest-free HOF-1a and guest-loaded HOF-1 and exhibits reversible pore contraction/expansion behavior.

The process of pore expansion/contraction can be effectively characterized by *in situ* PXRD of the gas-loaded sample. In 2023, our group reported a flexible-robust HOF (HOF-FJU-8) derived from 4,4′,4′′,4′′′-(pyrrolo[3,2-*b*]pyrrole-1,2,4,5-tetrayl)tetrabenzonitrile (DP-4CN) by employing a sticked-layer strategy.^43^ Based on the CO_2_ sorption isotherm at 196 K, the activated HOF-FJU-8a exhibits a stepwise adsorption isotherm, suggesting its framework flexibility and the existence of a gating effect. Furthermore, *in situ* PXRD of CO_2_-loaded HOF-FJU-8a at 196 K confirmed that with pressure increasing, Miller indices (102̄) shift slightly to the right, indicating a slight change in the structure. The pore-size distribution calculated from the CO_2_ isotherm is also slightly larger than the calculated one based on the single crystal structure due to the pore space expansion during the third step of the adsorption process.

#### 2.1.2 Structural deformation

**Fig. 5:**
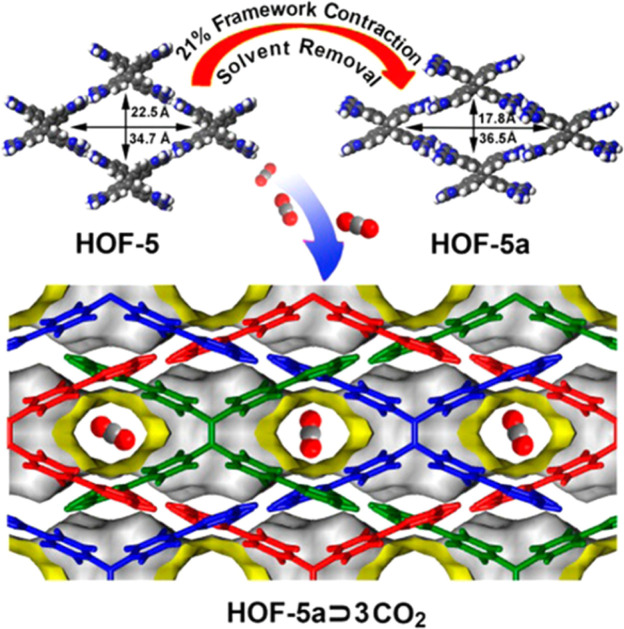
Illustration of the structural deformation of HOF-5 and the arrangement of CO_2_ molecules in the pores.^45^

This flexible behavior is more common in MOFs since the flexibility originating from the metal nodes can easily cause changes in the coordination angle, leading to deformation of the channels, *e.g.*, the well-known MIL-53 (Cr and Al).^74–76^ In HOFs, the structural deformation can come from the flexibility of the organic units or H-bonds. For example, in HOF-5, which is constructed from 4,4′,4′′,4′′′-tetra(2,4-diamino-1,3,5-triazin-6-yl)tetraphenylethene (DAT), planar 4-connected organic building units can undergo notable structural deformation after full activation (Fig. 5).^45^ The activated sample HOF-5a retains the same framework connectivity and topology but has less pore volume (41.1%) than HOF-5 (55.3%). The size of the channel in HOF-5 (22.5 × 34.7 Å) contracts to 17.8 × 36.5 Å in HOF-5a, which means that the shape of the channel is getting flattened.

Similar flexible behavior has been reported in other HOFs as well.^46–48^ In 2021, Chi *et al.* reported the design and synthesis of a dynamic two-dimensional (2D) woven HOF.^48^ The researchers were able to emulate a weaving craft by interlocking 1D strands through H-bonding, resulting in a 2D molecular woven network. Thus, the dynamic nature of the woven structure allows for reversible structural deformations in response to different solvents, as well as large-scale elasticity switching.

### 2.2 Interpenetration/stacking manner

It has been established that interpenetration is a crucial structural characteristic of porous materials because it can increase structural stability, endow the framework with flexibility, and allow for fine-tuning of the pore structure.^77,78^ Therefore, realizing a controlled interpenetration design in HOFs can enhance functional diversity. Similarly, the layer-stacked 2D HOFs can also exhibit flexible behavior due to the relatively weak π–π interactions between layers, resulting in sliding.

#### 2.2.1 Interpenetrated network

**Fig. 6:**
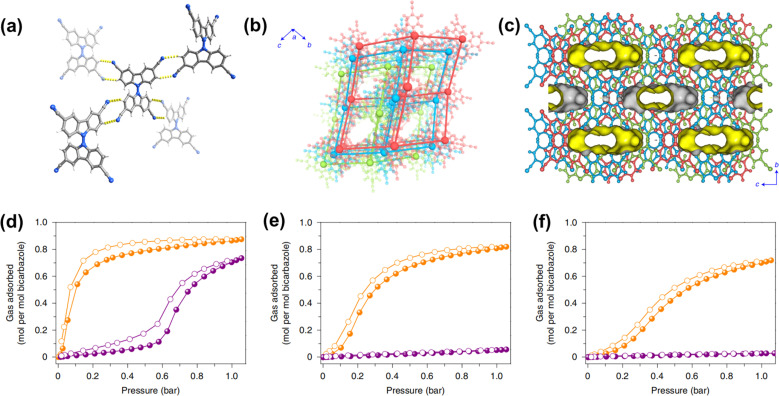
(a) The intermolecular H-bonding interactions between bicarbazole units; (b) the threefold-interpenetrated **dia** net; (c) pore channels along the [100] direction; (d–f) gas adsorption isotherms for C_2_H_4_ (orange) and C_2_H_6_ (purple) at 298 K, 318 K and 333 K, respectively.^52^

In 2021, our group reported a microporous HOF-FJU-1, which is composed of 3,3′,6,6′-tetracyano-9,9′-bicarbazole *via* intermolecular H-bonding interactions.^52^ Each bicarbazole unit is linked to four adjacent bicarbazoles by four pairs of C–N⋯H–C H-bonds with distances of 3.431–3.536 Å to form a **dia** topology (Fig. 6). HOF-FJU-1 exhibits a threefold-interpenetrated structure, with distinct offset π–π interactions along the *a* axis. The pore windows in the channels have a size of about 3.4 × 5.3 Å^2^, which is suitable for the separation of C_2_H_4_. This flexible HOF exhibits a gate-opening effect, where the opening pressure required for C_2_H_4_ uptake varies with temperature. The *in situ* PXRD patterns of the CO_2_ loaded sample at 195 K confirm the minor expansion of the pore window. Additionally, the interpenetrated network exhibits high stability even under various harsh conditions. A similar phenomenon can also be found in other cases.^49–51,53^

#### 2.2.2 Layer network sliding

**Fig. 7:**
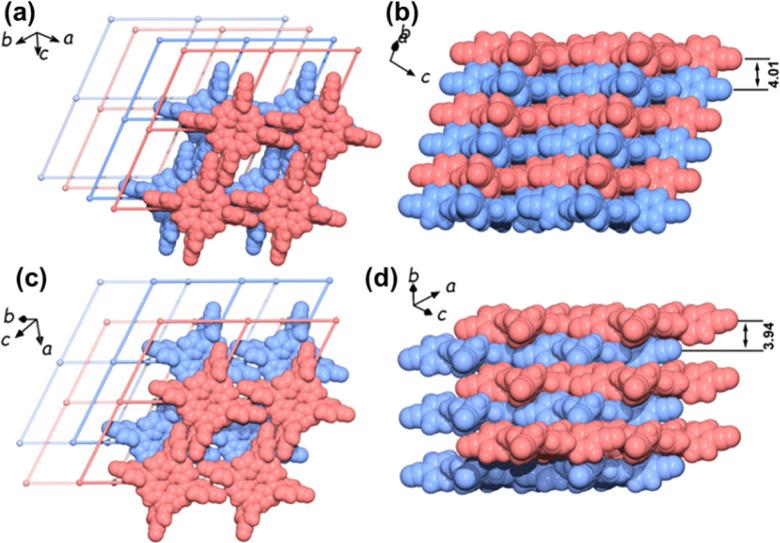
The AA stacking pattern of HOF-29 viewed perpendicularly (a) and side views (b); the AB stacking pattern of HOF-29⊃pX viewed perpendicularly (c) and side views (d).^57^

The flexible behavior of 2D HOFs has also been observed in single-crystal structures. In 2022, our group reported an adaptive HOF, HOF-29, based on 4,4′,4′′,4′′′-(porphyrin-5,10,15,20-tetrayl)tetrabenzonitrile (PTTBN).^57^ Each PTTBN unit is connected with four adjacent units *via* four pairs of intermolecular C–H⋯NC H-bonds, resulting in a 2D **sql** net. Different layers were packed in an AA stacking pattern through multiple H-bonding and π–π stacking interactions (Fig. 7a and b). HOF-29 exhibited a single-crystal-to-single-crystal transformation from the as-synthesized AA stacking phase to another AB stacking phase after adsorbing pX molecules by sliding the 2D layers and the local distortion of the ligand (Fig. 7c and d) realized the exclusive recognition of pX over mX, oX and EB.

In some other studies, the flexibility of 2D HOFs has already been applied in the field of gas separation.^56,58^ In 2023, Zhang *et al.* reported a CO_2_-selective HOF-FJU-88 from 2,4,6-tri(1Hpyrazol-4-yl)pyridine (PYTPZ) building units.^58^ HOF-FJU-88 consists of a 2D H-bonded layer formed from PYTPZ molecules. Each layer is further connected by π–π interactions between pyridine and pyrazole groups. During the activation process, PXRD indicates a partial loss of crystallinity due to the sliding of the 2D layers. The CO_2_ adsorption isotherm also exhibits a significant gate-opening effect, demonstrating the dynamic framework nature of HOF-FJU-88.

### 2.3 H-bond breaking/reconstruction

The reversibility of the H-bonds offers HOFs a unique type of flexibility; that is, HOFs are able to return to their initial states if the structures collapse following a stimulus response, which includes physical stimuli (pressure, temperature, guest molecules, *etc.*) and chemical stimuli (acid, base, corrosive gases, *etc.*).^59–69^ Or, sometimes, the H-bonding patterns will just be reorganized without losing the framework crystallinity, thus leading to a structural transformation. For example, the ethanol-bridged carboxyl dimers in HOF-30 reported by Wang *et al.* have exhibited reversible structural transformations with the removal of ethanol during the activation process or soaking in the solvent again.^69^

**Fig. 8:**
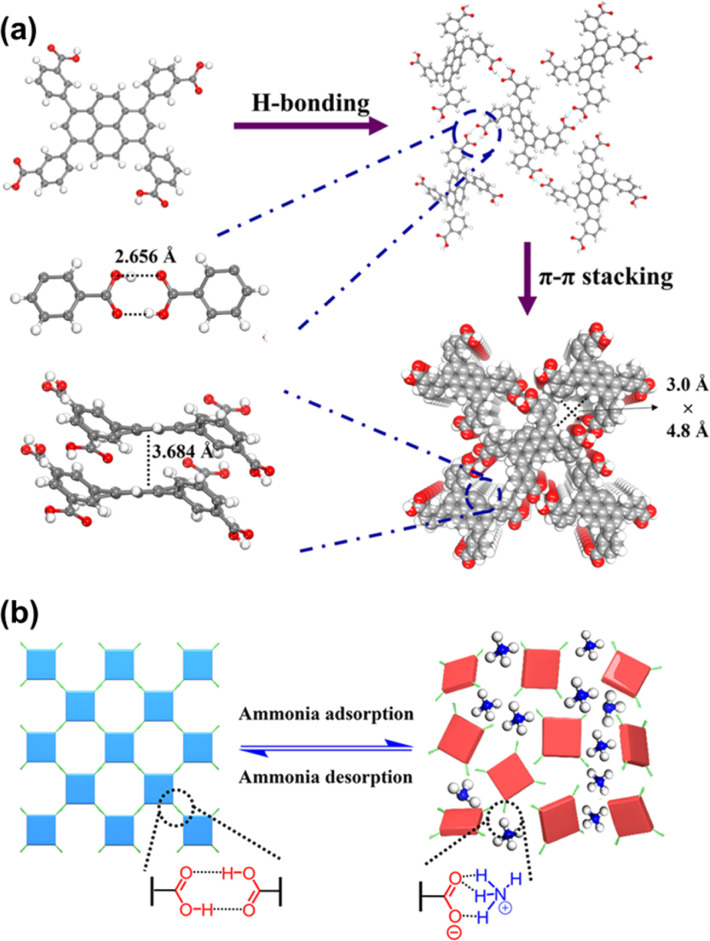
(a) Crystal structures of FDU-HOF-3; (b) schematic illustration of the self-healing effect of FDU-HOF-3 before and after NH_3_ capture.^64^

In 2024, Li *et al.* reported a microporous HOF, FDU-HOF-3, for ammonia (NH_3_) capture. This HOF is developed from 3,3′,3′′′,3′′′′–(pyrene-1,3,6,8-tetrayl)tetrabenzoic acid (H_4_PTTB) organic units.^64^ In the structure, each H_4_PTTB building block was connected to four adjacent units *via* intermolecular H-bonds, along with intermolecular π–π stacking interactions, forming a three-dimensional (3D) network with **sql** topology (Fig. 8a). Interestingly, FDU-HOF-3 will lose its crystallinity after NH_3_ adsorption. However, after regeneration by heating and degassing, the regenerated HOFs return to the crystalline phase again. Further research demonstrated that the COOH–NH_3_ acid–base interactions, along with the breaking and regeneration of COOH–COOH H-bonds, contributed to this HOF's self-healing behavior (Fig. 8b).

**Fig. 9:**
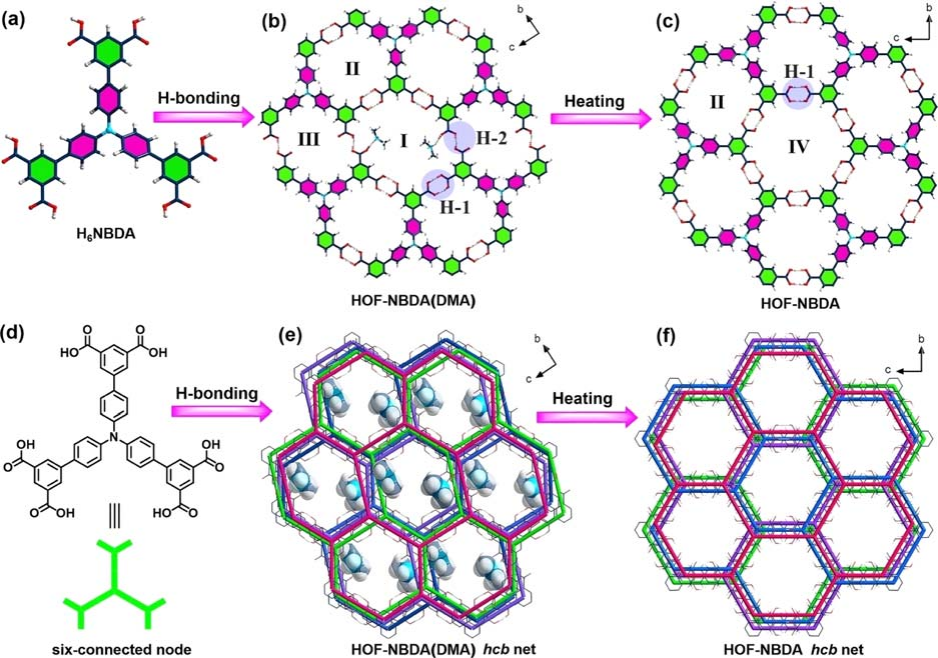
(a) The structure of H_6_NBDA; (b and c) representation of the reorganization of H-bond dimers upon heating; (d) scheme of H_6_NBDA and the six-connected node; (e and f) the transformation of layered frameworks before and after heating.^67^

Another typical example was reported in 2023 by Wu *et al.* HOF-NBDA(DMA) was constructed from 4′,4′′,4′′′-nitrilotris([1,1′-biphenyl]-3,5-dicarboxylic acid) (H_6_NBDA).^67^ Each organic unit is connected to three adjacent H_5_NBDA units through six sets of intermolecular H-bonds to form a 2D honeycomb-like layer. It is worth noting that there are two types of H-bonds in HOF-NBDA(DMA). As shown in Fig. 9, H-1 is the classic carboxyl dimer that forms a pair of parallel H-bonds. However, in H-2, one carboxyl group was deprotonated, and thus further connected to another DMA cation *via* a N–H⋯O H-bond to balance the charge, resulting in a relatively low symmetry of the monoclinic *P*1̄ space group. However, upon activation, HOF-NBDA(DMA) transformed into another structure, HOF-NBDA. The single-crystal structure revealed that only H-1 type H-bonds remain; all the carboxyl groups are not deprotonated. The resulting HOF-NBDA exhibited a relatively high symmetry of the orthorhombic *Fddd* space group.

**Fig. 10:**
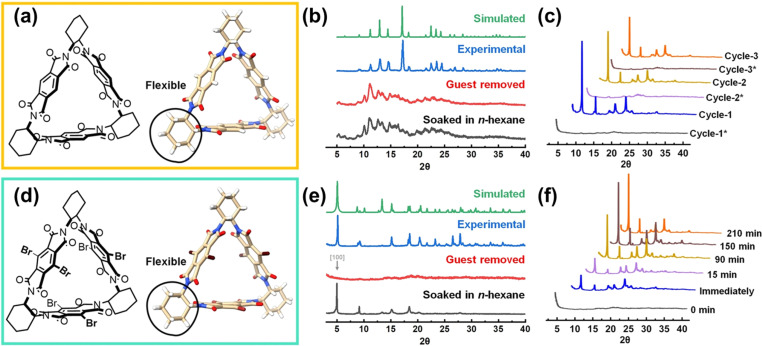
(a and d) The chemical structures of Tri-PMDI and Tri-PMDI-Br, respectively; (b) PXRD patterns of PMC-Tri-PMDI; (c) PXRD patterns of HOF-Tri-PMDI-Br after removal and resoaking in *n*-hexane three times; (e) PXRD patterns of HOF-Tri-PMDI-Br; (f) PXRD patterns of HOF-Tri-PMDI-Br soaked in *n*-hexane for different intervals of time.^62^

In 2023, Zhao *et al.* reported a very interesting study. Two different macrocyclic molecules, trimeric pyromellitic diimide (Tri-PMDI) and Tri-PMDI-Br, are synthesized and crystalized into two distinct structures (Fig. 10).^62^ In Tri-PMDI, H-bond interactions between the Ph–H and the O=C hold the molecules together and finally lead to the formation of PMC-Tri-PMDI (PMC, porous molecular crystals). For Tri-PMDI-Br, an unusual H-bond forms between C(sp^3^)–H from the cyclohexane and O=C, leading to the formation of HOF-Tri-PMDI-Br. The obtained HOF-Tri-PMDI-Br can simultaneously exhibit robustness and flexibility. The structural flexibility enables a reversible transformation between non-crystalline and crystalline phases by introducing or removing some specific solvent molecules, which act as a “key” to control the crystallinity. The single crystal structure of *n*-hexane@HOF-Tri-PMDI-Br has shown that *n*-hexane could easily go through the smaller pore of HOF-Tri-PMDI-Br and form H-bonds with cyclohexane linkages. Interestingly, only the solvent molecules with short chains, such as *n*-pentane and *n*-hexane, have the effect of crystallinity recovery. From this example, we can see the unexpected impact that a cleverly designed organic building block can have on the properties of a flexible HOF.

**Fig. 11:**
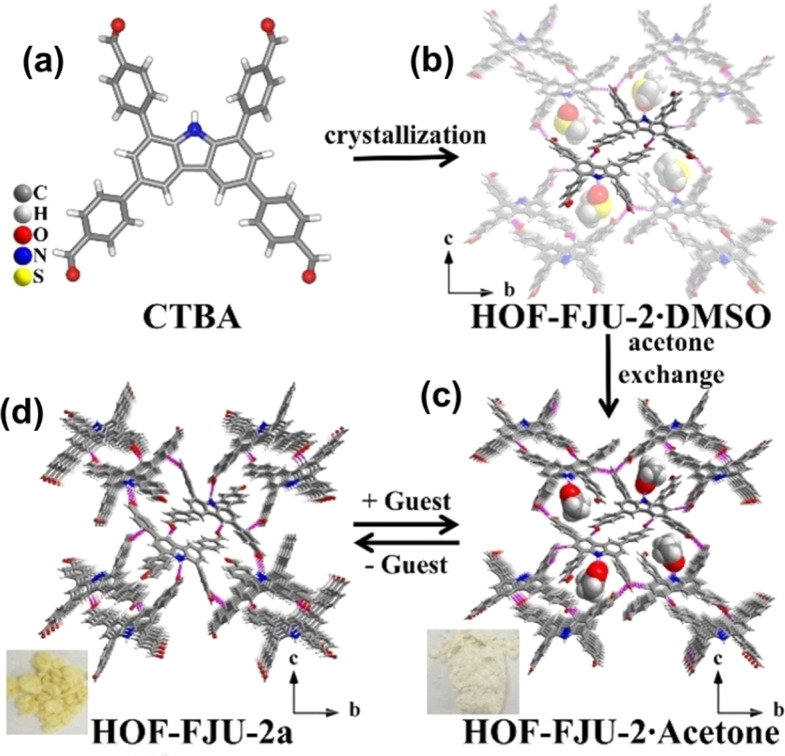
(a) The tetrabenzaldehyde molecule CTBA; (b) structure of HOF-FJU-2·DMSO; (c) HOF-FJU-2·Acetone; (d) reversible SCSC transformation after guest removal to nonporous HOF-FJU-2a.^66^

Recently, Zhang *et al.* reported a multifunctional HOF (HOF-FJU-2) using a tetrabenzaldehyde molecule, 4,4′,4′′,4′′′-(9*H*-carbazole-1,3,6,8-tetrayl) tetrabenzaldehyde (CTBA), with a carbazole N–H binding site (Fig. 11).^66^ This D–π–A type molecule has been confirmed to be able to construct flexible and/or flexible-robust HOFs for unprecedented functions. In this work, HOF-FJU-2 can be crystallized from different solvents. HOF-FJU-2 exhibits a 3D framework where CTBA molecules form π-stacking rod dimers. These dimers connect *via* H-bonds (C–H⋯O) and π–π interactions between aldehyde and carbazole groups, leading to the formation of larger π-stacking rod structures. The framework extends further through interactions between adjacent rod dimers, facilitated by C–H⋯O=C H-bonds. Interestingly, the activation of HOF-FJU-2 will result in a single-crystal-to-single-crystal (SCSC) transformation to a closed framework HOF-FJU-2a. However, when soaked in acetone or exposed to acetone vapor, the yellow HOF-FJU-2a crystals can be facilely transformed back into the white porous HOF-FJU-2, exhibiting flexibility in structure. Unlike HOF-FJU-1, the H-bonds formed between the CTBA molecules are not in pairs like in HOF-FJU-1, thus further increasing the local flexibility and leading to structural transformation during the activation process. However, the overall stronger intermolecular interactions still maintain the stability of HOF-FJU-2, allowing this flexible HOF to exhibit related properties while maintaining crystallinity.

### 2.4 Local dynamic behavior

The concept of local dynamics highlights the local motions throughout the entire framework in contrast to the behavior of global dynamics. It has been well established that modifying dangling groups on organic ligands in MOFs can lead to flexible behavior, even though the framework may not exhibit significant flexibility. The dangling groups can be small groups or complex interlocking supramolecular structures. Similarly, in HOFs, local flexibility in the organic units can bring about significant dynamic behavior, and this effect can either affect the properties or bring about a structural change. In addition, the rotation of the covalent bonds in the organic unit also brings about flexibility and results in structural diversity.^71–73^

**Fig. 12:**
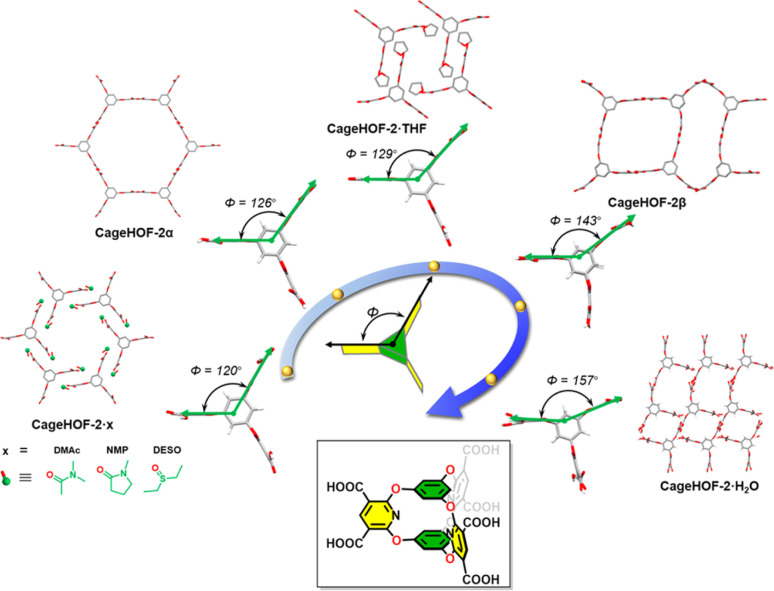
Schematic illustration of the dihedral angles of the pillar ring changes from 90° to 157° in Cage-6-COOH based structures.^73^

In 2023, Chi *et al.* reported a flexible HOF, 8PZ, with local dynamics for adaptive guest accommodation through incorporating soft ethyl-ester chains.^72^ 8PZ was developed from a flexible building block tetraethyl 4′,4′′′,4′′′′′,4′′′′′′′(ethene-1,1,2,2-tetrayl)tetrakis([1,1′-biphenyl]-4-carboxylate) (TPE-4PZ), which includes four identical soft ethyl-ester chains. 8PZ has been confirmed to have significant local dynamics in response to solvents and temperature changes, especially the in-plane and out-of-plane motions of terminal carbon atoms, leading to an effective approach to regulating the pore sizes. In 2023, Little *et al.* reported a flexible oxygen-bridged prismatic organic cage molecule, Cage-6-COOH, which has three pillars that exhibit rotational motion like a hinge in the solid state (Fig. 12).^73^ This organic building unit can form a series of flexible HOFs by crystallizing in different solvents. CageHOF-2α was crystallized from the THF/CH_3_CN solution, and each Cage-6-COOH molecule was connected to six neighboring Cage-6-COOH molecules *via* H-bonds between directional carboxylic acid dimers with distances of 2.58–2.59 Å. Although CageHOF-2α shows an **acs** topology, it is nonporous. Another CageHOF-2β was crystallized from ethanol with similar H-bonding patterns. However, in CageHOF-2β, the H-bond building units are not all planar, and the aromatic pillars have profoundly different orientations. CageHOF-2β exhibited good thermal stability and a BET surface area of 458 m^2^ g^−1^. Furthermore, another five HOFs are found with different dihedral angles between the pillars varying from 90° to 157°. The flexibility of Cage-6-COOH allows this molecule to rapidly transform from a low-crystallinity solid into CageHOF-2α and CageHOF-2β under mild conditions simply by using acetonitrile or ethanol vapor, respectively. This work highlights the potential of flexible organic cage hinges in the design and synthesis of flexible HOFs with tunable properties.

## 3. Diverse applications for flexible HOFs

Flexible HOFs stand out because of their inherent flexibility, offering a wide range of potential applications. Although still relatively short in development compared to flexible MOFs and COFs, flexible HOFs have demonstrated their unique advantages in gas storage/separation, molecular recognition, biomedicine, heterogeneous catalysis, chemical sensing, and other areas. Their outstanding flexibility, along with the reversibility of H-bonding, allows them to be synthesized from a diverse range of building blocks, enabling mild synthetic reactions, excellent solution processability, easy repair, easy regeneration, and recyclability.

### 3.1 Gas storage/separation

The ability of flexible HOFs to adjust their structure dynamically when facing changes in temperature, pressure, or guest molecule adsorption offers excellent potential for gas storage/separation. This dynamic response allows for interesting changes in pore sizes and shapes to effectively adsorb and separate specific gas molecules.^27,28,38,40–43,45,46,49,51–54,58,60,64,66,67,79–89^ In addition, the recyclability of HOFs reduces the cost. The dynamic behavior of flexible HOFs in gas adsorption makes the research more complex than that of other rigid porous materials. Therefore, many *in situ* characterization methods have been used in recent years to help us better understand this behavior.

Early in 2010, Schröder *et al.* reported the gas adsorption of SOF-1, which demonstrated impressive adsorption capabilities for CH_4_ (106 cm^3^ g^−1^, 10 bar, and 195 K), C_2_H_2_ (124 cm^3^ g^−1^, 1 bar, and 195 K), and CO_2_ (69 cm^3^ g^−1^, 16 bar, and 298 K).^28^ Most notably, the activated SOF-1a demonstrated a flexible response to temperature increases from 77 K to 125 K, with a significant increase in N_2_ adsorption capacity, indicating a certain framework flexibility. Such a flexible behavior appears to be more pronounced in HOF-1 and is applied for the first time in the field of gas separation.^27^ As described above, HOF-1 exhibited a significant gate-opening effect on C_2_H_2_. The adsorption isotherm of C_2_H_2_ showed a sudden increase around 200 mmHg, with a final adsorption capacity of 63.2 cm^3^ g^−1^ (at 800 mmHg and 273 K). Meanwhile for C_2_H_4_ it only adsorbed 8.3 cm^3^ g^−1^ under the same conditions, thus leading to a highly selective adsorptive separation of C_2_H_2_ and C_2_H_4_ at ambient temperature. The subsequently reported HOF-5 also exhibits high porosity and CO_2_ adsorption capacity.

After these early studies, a large number of flexible HOFs have been reported for gas adsorption/separation. As mentioned above, HOF-FJU-1 showed a clear gate-opening effect due to the interpenetrated framework, and the experimental results showed that the adsorption capacity for C_2_H_4_ was 47 cm^3^ g^−1^ at 298 K and 1 bar, while for C_2_H_6_ it was very low.^52^ In addition, with the increase in temperature, the adsorption capacity for C_2_H_6_ will further decrease. Breakthrough experiments confirmed the high selectivity of HOF-FJU-1 for C_2_H_4_ with a purity of 99.1% at 333 K. It is worth mentioning that HOF-FJU-1 exhibits stability under various harsh conditions and can be easily processed into different forms for gas separation. Interestingly, theoretical simulation shows that neither C_2_H_4_ nor C_2_H_6_ molecules can diffuse into HOF-FJU-1 without considering the flexibility of the framework, but the distribution of C_2_H_4_ in the pore is clearly observed by single crystal X-ray diffraction, which fully demonstrates the key role of flexibility in this adsorption process. However, in another study, instead of showing the same flexibility towards smaller gas molecules (C_2_H_2_ and CO_2_), HOF-FJU-1 achieves the high sieving effect of C_2_H_2_/CO_2_.^54^ Therefore, such an interesting robust-flexible HOF exhibited completely different adsorption behaviors with different adsorption guests, showing the remarkable versatility of flexible HOFs.

**Fig. 13:**
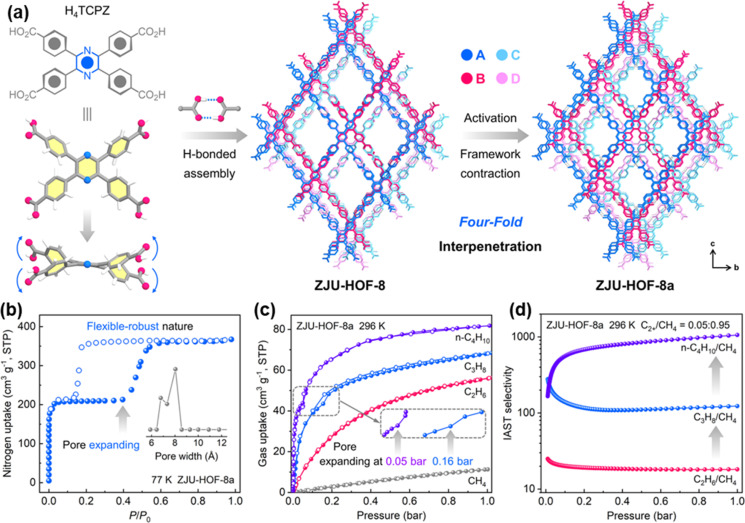
(a) Single-crystal structure of ZJU-HOF-8 and the structural transformation with framework contraction upon activation; (b) N_2_ adsorption of ZJU-HOF-8a at 77 K; (c) different gas adsorption at 296 K; (d) IAST selectivities of ZJU-HOF-8a at 296 K.^55^

Very recently, Li *et al.* reported a carboxyl-based HOF, ZJU-HOF-8, with flexible-robust porosity for natural gas purification.^55^ ZJU-HOF-8 is constructed from 2,3,5,6-tetrakis(4-carboxyphenyl)-pyrazine (H_4_TCPZ) organic units *via* intermolecular C–H⋯N, C–H⋯O and C–H⋯π interactions (Fig. 13a). ZJU-HOF-8 exhibits a unique four-fold interpenetrated subnetwork. Gas sorption and X-ray diffraction studies confirm that the activated ZJU-HOF-8a exhibits a significant structural contraction, showing selective pore-pocket opening by certain gas molecules. This selective opening leads to higher uptakes and enhanced selectivities for C_3_H_8_ and *n*-C_4_H_10_ over C_2_H_6_ and CH_4_, highlighting the potential for designing flexible-robust HOFs for improved gas separation (Fig. 13b–d).

The potential application of flexible HOFs in gas adsorption/separation is actually more widespread. The renewability of HOFs, in particular, provides this material with a unique economic advantage. Their lower density, on the other hand, allows for a better balance of volumetric and gravimetric uptake, which is also an important aspect in this field.^90^ Flexible HOFs, in general, can exhibit more unpredictable dynamic behaviors, allowing them to deal with some difficult separation systems and establish a new class of energy-saving and environmentally friendly physical adsorbents.

### 3.2 Molecular recognition

In molecular recognition applications, flexible HOFs can precisely accommodate guest molecules within their pores through dynamic structural changes.^47,57,86,91^ This attribute enables selective binding and recognition of target molecules, which is crucial for sensors, drug delivery systems, and chemical sensing applications. By adjusting the framework's flexibility, researchers can enhance the specificity and efficiency of molecular recognition processes, paving the way for advanced sensing technologies and targeted therapeutics.

Ward *et al.* have reported a series of host frameworks based on guanidinium cations and interchangeable organosulfonate anions, which exhibited impressive flexible behaviors. In these compounds, the guanidinium molecules are connected to sulfonate *via* N–H⋯O–S H-bonds, forming a 2D quasi-hexagonal H-bonding network.^25,92^ Different types of organosulfonate molecules will further connect the layer, resulting in diverse structures. The flexibility of the H-bonded network allows these compounds to adapt to changes in the steric requirements of guest molecules that occupy the channels. The typical example reported in 1997 described a case of HOF, (G)_2_(BPDS) based on guanidinium (G) and 4,4′-biphenyldisulfonate (BPDS).^25^ Single-crystal diffraction results show that each BPDS molecule is connected to six G molecules *via* N–H⋯O–S H-bonding, resulting in a pillared structure. A variety of flexible response forms are observed, including (1) N–H⋯O–S H-bonding rotations; (2) twisted C–C bonding in BPDS molecules; (3) rotations of the C–S bonding in the BPDS molecules; (4) different H-bonding patterns in the guanidinium-sulfonate layer structure; (5) selection of “bilayer” *versus* “brick” stacking patterns. Guanidinium is capable of changing its pore structure to accommodate various monosubstituted benzene guests and disubstituted isomers, revealing host–guest interactions.

**Fig. 14:**
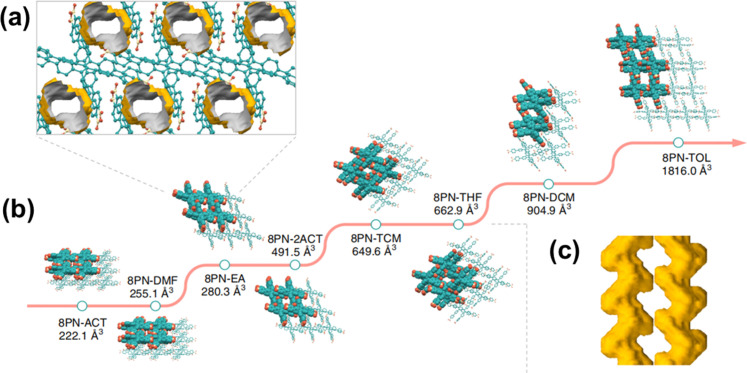
(a) Packing model of 8PN-EA in the [010] direction, with the solvent-accessible void space; (b) single-crystal X-ray structures of eight 8 PN frameworks with varying void space; (c) S-shaped channel surface of 8PN-THF.^47^

Another interesting recent study was reported in 2019. Chi *et al.* found an exceptionally flexible HOF, 8 PN, which exhibited permanent porosity derived from 1,1,2,2-tetrakis(4′-nitro-[1,1′-biphenyl]-4 yl)ethane.^47^ 8 PN exhibits unprecedented flexibility, allowing for the regulation of pore volume in HOFs through the control of molecular assembly and conformation. Nine different single crystals of 8 PN achieved a pore volume adjustment ranging from 89.4 Å^3^ to 1816.0 Å^3^ (Fig. 14). Moreover, the pore volume adjustment enables multimodal reversible structural transformations in response to various external stimuli, including guest molecules, temperature variations, and mechanical pressure changes. Flexible frameworks can accommodate guest molecules of different sizes, thereby yielding five high-quality co-crystals, further indicating the potential application prospects of flexible 8PN in adaptively regulating pore structures.

**Fig. 15:**
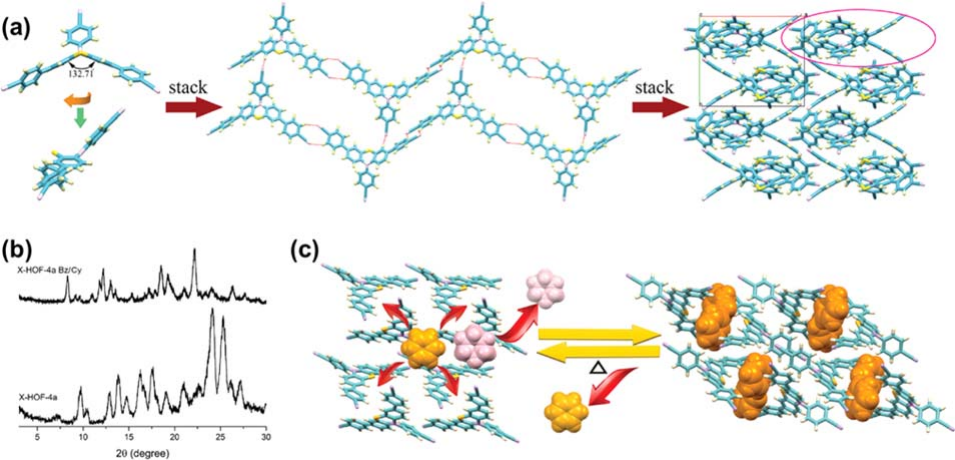
(a) Assembling of PTTCN molecules (left) to a 2D H-bonded framework with pores (middle), and intermolecular stacking in a 2 × 2 × 2 cell (right); (b) PXRD patterns; (c) schematic representation of the separation for benzene/cyclohexane.^93^

In 2023, Xue *et al.* reported a flexible luminescent HOF for the separation of benzene and cyclohexane.^93^ The researchers designed and synthesized a nonplanar phenothiazine derivative with three cyano moieties (PTTCN) as the functional crystal (Fig. 15). Two different forms of PTTCN crystals, ax form and eq form, have different fluorescence colors. The ax form crystals were found to selectively adsorb benzene through an SCSC transformation. However, the purity of the separated benzene from a benzene/cyclohexane equimolar mixture was relatively low at 79.6%. On the other hand, the eq form of PTTCN molecules co-assembled with benzene to construct an HOF (X-HOF-4) with S-type solvent channels and yellow-green fluorescence. This flexible HOF exhibited a strong preference for aromatic benzene over cyclohexane. The researchers discovered that the framework could release benzene to form a nonporous guest-free crystal under heating. This nonporous crystal could selectively reabsorb benzene from the benzene/cyclohexane mixture, allowing for the recovery of the original framework. The purity of the reabsorbed benzene reached approximately 96.5%. This work addressed the challenge of separating benzene and cyclohexane by designing a flexible luminescent HOF with selective adsorption and release properties.

### 3.3 Sensing

HOFs have demonstrated significant promise in the fields of multiple stimulus-response and intelligent optics because of their modular building units and flexible frameworks. As a multifunctional luminescent framework material with both luminescent and porosity properties, it has a broad application prospect in the field of sensing.^63,68,72,94–102^

**Fig. 16:**
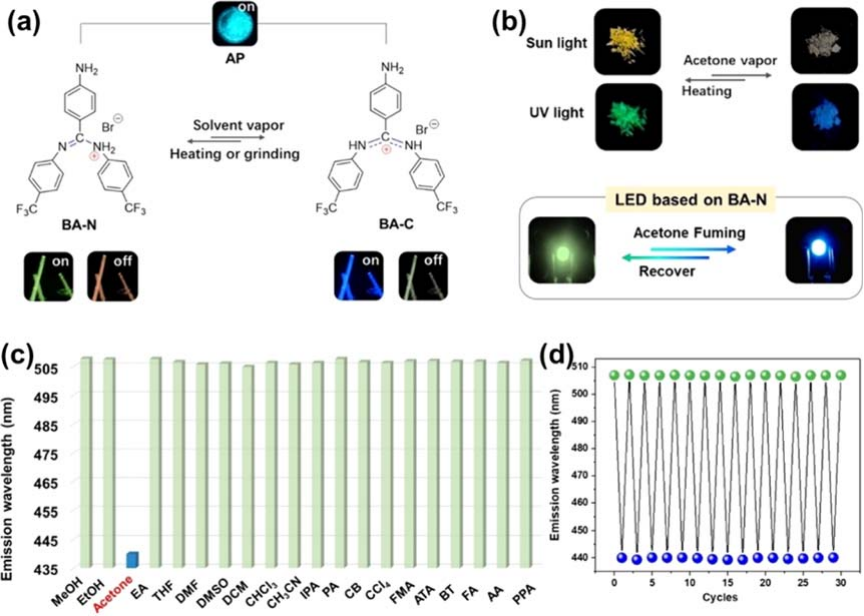
(a) Tautomerism of BA-N and BA-C; (b) images showing reversible switching between BA-N and BA-C (*λ*_ex_ = 365 nm), as well as LED light-emitting images before and after acetone fumigation; (c) histogram of the prompt emission peaks of BA-N following fumigation; (d) repeatability of luminescence color switching when stimulated with acetone vapor and heated.^68^

A novel triaryl formamidine salt containing two isomers (BA-C and BA-N) was reported by Lin *et al.*^68^ These two isomers can be recrystallized in different solvents to form two different HOFs, and it was found that the removal of acetone from the lattice of BA-C by grinding or heating could lead to the conversion of BA-C to BA-N, and in turn, exposing to acetone vapor or cooling at 77 K could lead to the conversion of BA-N to BA-C, thus realizing the reversibility of the conversion of BA-N and BA-C, and showing the flexibility of these HOFs (Fig. 16). The flexible behavior of this anionic HOF enables dynamic switching of multiple luminescence behaviors, including prompt fluorescence, TADF, and phosphorescence. As a result, this HOF can be used for highly sensitive and specific sensing of acetone with an ultra-low detection limit of 66.74 ppm.

There are many similar examples of utilizing the flexible behavior of HOFs to achieve fine-tuning of optical properties, such as the previously mentioned 8PZ, where soft ethyl-ester chains bring adaptability to different guest molecules, and endow it with programmable temperature-dependent luminescence behaviors.^72^ In another case, Cong *et al.* reported a novel polycatenated HOF MEP-HOF, which is composed of fluorenylidene-aza[1_6_]cyclophane (FLAC).^63^ This HOF exhibits dynamic reversible transformation in crystallinity during guest removal/adsorption and also exhibits sensitive detection of nitrobenzene. In 2021, Xue *et al.* investigated the influence of guest molecules on the photoluminescence and force-stimuli response of X-HOF-1.^102^ The research demonstrated that the mechanofluorochromic HOF materials could be regenerated through recrystallization and adsorbing the guest and highlighted the potential of guest molecules in regulating the properties and functions of flexible HOFs.

### 3.4 Biomedical applications

HOFs are a promising material for biomedical applications due to their excellent biocompatibility and low toxicity. One of the most significant advantages of flexible HOFs is their ability to adaptively accommodate a variety of guest molecules, enabling reversible guest encapsulation and controlled release in response to mild stimuli. Thus, flexible HOFs offer promising opportunities for targeted drug delivery, antimicrobial therapies, and anticancer treatments, providing innovative solutions to biomedical challenges.^103–106^

**Fig. 17:**
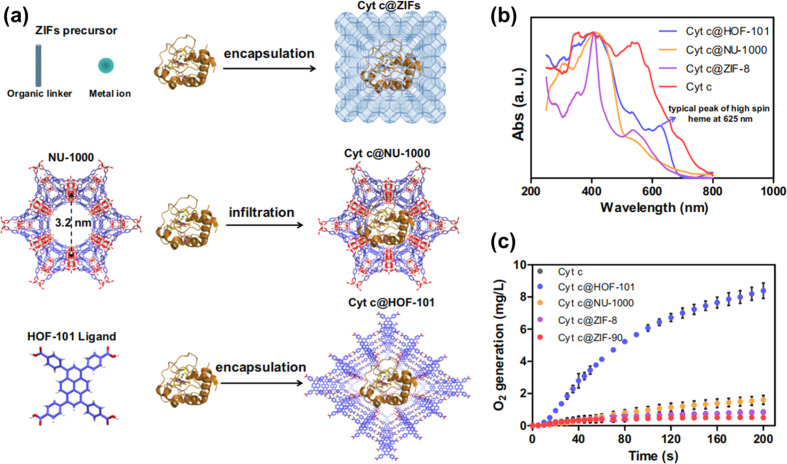
(a) Schematic illustration of the synthesis of Cyt c@ZIFs, Cyt c@NU-1000, and Cyt c@HOF-101; (b) normalized UV-Vis DRS; (c) CAT-like bioactivities of Cyt c@ZIFs, Cyt c@NU-1000 and Cyt c@HOF-101.^108^

A very typical example of a drug carrier is PFC-1, which is a photoactive HOF for synergetic chemo-photodynamic therapy.^107^ Subsequently, much work has been reported in this field, and flexible HOFs act as carriers or shells to protect or transport biomedical molecules. In 2022, Ouyang *et al.* reported a case where by encapsulating cytochrome c (Cyt c), a heme-containing enzyme, within HOF-101, they could create a biomimetic system that allowed for non-native biocatalytic activity (Fig. 17).^108^ The results of the experiment demonstrate that the H-bonded nano-biointerface between encapsulated Cyt c and the HOF cage induces Cyt c to alter its native conformation. This work demonstrates the synergistic relationship between Cyt c and HOFs in terms of structural flexibility.

**Fig. 18:**
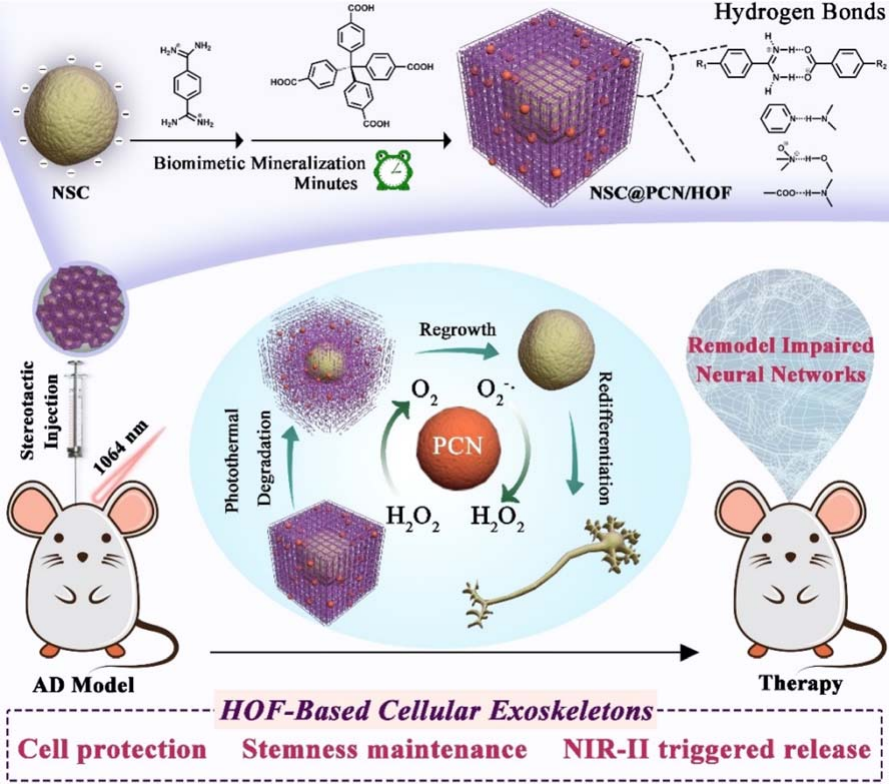
A schematic representation of the construction and removal of HOF shells on individual NSCs, as well as the process for remodeling impaired neural networks.^109^

In recent years, more and more scientists have applied flexible HOFs in the biomedical field. In 2022, Qu *et al.* reported the development of a novel strategy for the encapsulation and transplantation of neural stem cells (NSCs) using a HOF as a protective shell (Fig. 18).^109^ The HOF-based cell protectors effectively shielded the NSCs from physical and chemical stressors. They were degradable under near-infrared II (NIR-II) laser irradiation, allowing for controlled release of the encapsulated NSCs, which benefited from the weak bonding strength of HOFs. The transplantation of NSC@PCN/RA/HOF and subsequent NIR-II laser irradiation resulted in significant improvement in cognitive function in AD mice, as demonstrated by the Morris water maze test. The treated mice showed shorter escape latency, increased time spent in the target quadrant, and improved swimming speed compared to the control groups. Overall, this research demonstrates the potential of HOF-based cell encapsulation and transplantation as a promising strategy for the treatment of impaired neural networks, particularly in the context of neurodegenerative diseases like Alzheimer's disease.

### 3.5 Proton conductivity

HOFs are excellent candidates for proton conductors due to their intrinsic H-bonding networks. In general, the structural diversity of HOFs allows for the construction of a well-defined H-bonding network in the structure, either in the host framework or from guest molecules, which enables proton motion. A large number of studies have demonstrated the advantages of HOFs as a platform for proton conductors.^110–116^ For example, in 2011, Kim *et al.* reported proton conductivity in organic molecular porous materials based on cucurbituril (CB) compounds.^115^ Among them, CB[6]·H_2_SO_4_ exhibited the highest proton conductivities at 98% RH and 298 K with a value of 1.3 × 10^−3^ S cm^−1^, representing one of the pioneering studies that demonstrated the application of HOFs in this area. Another important study reported by Ghosh *et al.* described two porous HOFs, HOF-GS-10 and HOF-GS-11, based on arene sulfonates and guanidinium ions. The materials show high proton conduction values of 0.75 × 10^−2^ S cm^−1^ and 1.8 × 10^−2^ S cm^−1^ under humidified conditions.^114^ Although the flexible behavior of HOFs in proton conduction is not always visible, the simplicity of processing and regeneration due to their flexibility also benefits this application.

In 2023, our group reported a 2D layered structure by constructing a donor–acceptor π–π stacked HOF (HOF-FJU-36) utilizing 1,1′-bis(3-carboxyphenylmethyl)-4,4′-bipyridine (H_2_L^2+^) as the acceptor and 2,7-naphthalene disulfonate (NDS^2−^) as the donor.^112^ The presence of three water molecules in the channel, interconnecting the acids by H-bonding, forms a 3D framework. Continuous π–π interactions along the *a*-axis direction and smooth H-bonding chains along the *b*-axis direction provide pathways for electron and proton transport. After 405 nm illumination, the photogenerated radicals were able to give HOF-FJU-36 both switchable electron and proton conductivity due to coupled electron-proton transfer. This work demonstrated that by rationally designing flexible HOFs, the coupling of proton-electron transfer can be realized, resulting in controllable photoresponsive electronic and proton conductivity.

### 3.6 Other applications

Further research on flexible HOFs remains ongoing, and their applications involve many areas that will not be detailed here, such as catalysis,^61,117–122^ chiral separation,^123,124^*etc.*

**Fig. 19:**
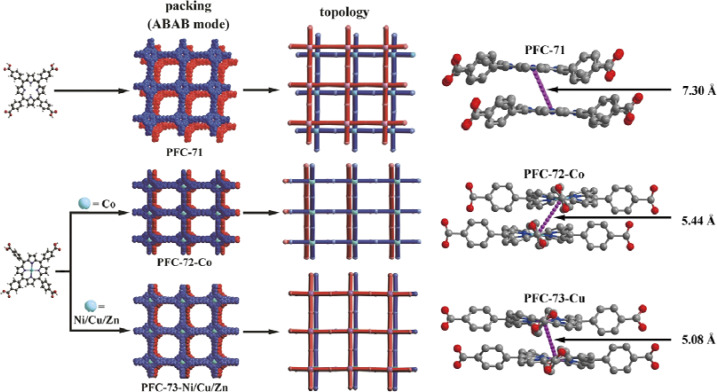
(left) Schematic representation of the structure and topology of PFC-71, PFC72-Co, and PFC-73-Ni/Cu/Zn; (right) the interlayer porphyrin center-to-center distance of PFC-71, PFC72-Co, and PFC-73-Cu.^61^

In 2022, Liu *et al.* reported the synthesis and characterization of porphyrin-based HOFs (PFC-71, PFC-72, and PFC-73) for photocatalytic CO_2_ reduction.^61^ [5,10,15,20-tetrakis(4-carboxyphenyl)porphyrin] (TCPP) is applied to synthesize these HOFs with different metalized porphyrin centers. In the case of PFC-71, the porphyrin center is not metalized, whereas, in PFC-72, PFC-73-Ni, PFC-73-Cu, and PFC-73-Zn, the porphyrin center is metalized with different metal ions (Co, Ni, Cu, and Zn, respectively). This metallization of the porphyrin center leads to a larger electronegativity difference on the macrocycle backbone, causing increased polarizability and electron cloud distortion, resulting in the formation of stronger offset π–π interactions between adjacent interlamellar porphyrins. PFC-72 and PFC-73 exhibit higher stability compared to PFC-71. The undulated geometry of the metalized layers, along with the deeper interlayer penetrations and orientation of benzene rings orthogonal to the layer, increases the geometrical barrier for sliding and contributes to the higher stability of PFC-72 and PFC-73 (Fig. 19). The authors investigate the metallization process of the HOFs and its effect on the photocatalytic activity, demonstrating the potential of these HOFs as photocatalysts for CO_2_ reduction.

### 3.7 Future directions for flexible HOFs' applications

Over the last decade, research on flexible HOFs has demonstrated great potential for diverse applications. In-depth research into the structure–function relationship has greatly improved the response of flexible HOFs to a broader range of stimuli, particularly in biomedical applications. In recent years, there has been a lot of interest in the development of HOF membranes, and flexible HOFs have the potential to expand the range of applications in this field even further.^125^ In addition, researchers are improving the synthesis and processing technologies of flexible HOFs in order to enable further application in industry. Overall, research in the future should concentrate on utilizing the unique features of flexible HOFs to provide inventive solutions for a variety of practical applications.

## 4. Conclusions and outlook

In this perspective, we have summarized and analyzed the very common and interesting phenomenon of flexibility in HOFs. We believe that flexibility refers to the ability or range of deformability of the overall or local structure, *i.e.*, the ability to undergo structural changes or deformations under certain stimuli without losing (or restoring) its crystallinity or function. This flexibility allows HOFs to be adapted to different environments and applications. We should note that the flexibility of HOFs differs notably from that of MOFs and COFs. First, the flexibility of HOFs mainly comes from the flexibility and reversibility of H-bonding. H-bonding is important for HOFs' flexible behavior, and they can be distorted, broken, and reformed in response to external stimuli, which is a unique property of HOFs. MOFs, on the other hand, are typically flexible due to their metal–organic coordination bonds between metal ions and organic ligands, which can undergo stretching or rotation to some extent, making MOFs flexible to a limited degree. COFs are composed of organic molecules connected by covalent bonds that can be bent or twisted under external stresses, allowing them some deformability. The distinctive features of flexible HOFs endow them with some special advantages, but they also pose challenges for their design and application.

Currently, the challenges of flexible HOFs mainly focus on: (1) the complexity of structural design; (2) framework stability; (3) controlling H-bond dynamic behaviour. Designing flexible HOFs requires precise control of the structure and arrangement of organic molecules to ensure that the framework can undergo reversible changes. However, flexible HOFs usually have lower structural stability due to the flexible and reversible nature of H-bonding, and thus, the relationship between flexibility and stability needs to be balanced in the design. At the same time, it is essential to explore how to effectively control the kinetic behaviour of H-bonds to achieve the desired structure change and performance.

HOFs are still facing challenges and opportunities in their applications. Although typically HOFs are not as stable as MOFs and HOFs in terms of their structure rigidity and thus porosity, some HOFs can indeed be very stable even under highly acidic/basic conditions and high temperatures, particularly when framework interpenetration and other weak interactions such as π⋯π and C–H⋯π can collaboratively reinforce the HOF framework. The simple recrystallization nature of most HOF materials can allow us to easily and straightforwardly regenerate HOF materials for their reusage, which will save the material costs for some of their applications, particularly in gas storage and separation, and catalysis. Apparently, HOFs are not as designable as MOFs and HOFs, so extensive exploration of different synthetic approaches is still necessary to discover some unique HOF materials for different applications. To make use of the framework flexibility, we can execute more parameters through changing the temperatures and pressures to finely tune and maximize the gas separation and purification, targeting some functional HOF materials for gas separation/purification even without our imagination. Because HOFs purely contain only organic species and can be reversibly dissociated/re-assembled, their compatibility with biological systems is expected to be better than that of MOFs and COFs. HOFs also do not have metal ion species, and this might be another advantage for HOFs for their biomedical applications. Given the fact that HOF structures can be easily stimulated even by some very weak external stimuli such as light and ultrasonic irradiation, HOFs are very promising materials for drug delivery and thus for the treatment of some challenging diseases. As fast development in MOF chemistry and materials science, HOF composite materials will be also developed in the near future for their broad applications. In conclusion, flexible HOFs will provide us with the bright promise to design and construct many HOF materials for a variety of applications. We believe that some flexible HOF materials might be eventually implemented in practical applications in energy and environmental science and biomedical applications in the future.

## Author contributions

J. L. and B. C. performed the literature search, analyzed the published results, and wrote the manuscript.

## Conflicts of interest

There are no conflicts to declare.
